# When self-harm is about preventing harm: emergency management of obsessive–compulsive disorder and associated self-harm

**DOI:** 10.1192/bjb.2020.70

**Published:** 2021-04

**Authors:** Erika Palombini, Joel Richardson, Emma McAllister, David Veale, Alex B. Thomson

**Affiliations:** 1 Central and North West London NHS Foundation Trust, UK; 2 Lived experience advisor, UK; 3 South London and Maudsley NHS Foundation Trust, UK; 4 King's College London, UK

**Keywords:** Self-harm, obsessive–compulsive disorder, consent and capacity, phenomenology, emergency psychiatry

## Abstract

Mental health staff may have limited exposure to emergencies associated with obsessive–compulsive disorder (OCD) during postgraduate training. The first time they encounter a person in the midst of severe obsessions, or one who has compulsively self-harmed in response to such obsessions, might be when working on call covering the emergency department. This educational article presents the lived experience of one of the authors as a clinical scenario. The scenario is then used to illustrate the severity of disability and the rates of self-harm and suicide-related mortality caused by OCD. The recognition and assessment of OCD is described, along with what helps in emergency situations. Written informed consent was obtained for the publication of clinical details.

## Scenario

### Doctor's perspective

You are the doctor on call for psychiatry. The emergency department registrar has referred a 37-year-old professional who is in resus with police in a state of extreme distress, with self-inflicted chemical burns from corrosive alkali on her arms and torso, and haemoptysis caused by the associated fumes. This is her 133rd attendance in 4 years. She is repeatedly saying, ‘It's all contaminated, I need to go’. A member of the crisis team has advised you to refuse the referral, because seeing the patient would reward her attention-seeking behaviour. When you access her records, there is a ‘yellow flag’ advance plan which states she attends the emergency department frequently and should not be seen by psychiatry.

### Patient's perspective

*I am the patient. I don't want to be here. The police restrained me, put me in a cage in the back of a van and brought me to hospital ‘voluntarily’, after finding me walking down the street coughing up blood on my hands and clothes. I'm terrified. I've been worrying for several days that I've hurt or killed people. I've had worries like this since primary school, but things have got worse again lately. Because I keep thinking I've killed someone, when the police stopped me I thought they were arresting me for murder. Now I'm in resus, I'm worried the other patients here are hurt because of me. I can feel contamination under my skin that is hurting people, and I have been trying to get it out. I used to use soaps and bleaches, but they didn't work, so I now use corrosive alkali to try to remove the contamination. I needed to go to the shop to buy more when the police stopped me. I'm scared the staff will get contaminated too, and I don't want them to touch me. They want me to take off my clothes, and they're holding me down to try to put a line in my arm, and I can't. I just need to go. I need to sort out the contamination and I don't want anyone else to be hurt*.

Question 1: How would you respond to the emergency department registrar?

(a) Refuse the referral because seeing the patient would reward her attention-seeking behaviour.
(b) Refuse the referral because there is a care plan saying the patient should not be seen.
(c) Accept the referral and go to assist the emergency department staff as soon as possible.
(d) Accept the referral and tell the emergency registrar that you will see the patient only after she has been ‘medically cleared’.

Answer: (c)

Best practice guidance is clear: people attending hospital after an episode of self-harm should all receive a biopsychosocial assessment by a clinician with adequate skill and experience, and this should not be delayed by waiting for medical treatment to be completed.^1–4^ Although the last health service contact for many people who die by suicide was with a general hospital, only around half of people attending emergency departments with self-harm receive a psychosocial assessment.^5,6^ Pejorative terms such as ‘attention-seeking’ persist in clinical practice; they do not justify the withholding of psychiatric assistance, may be related more to animosity and shaming directed by healthcare staff towards people who self-harm, and have no place in advance care planning.^7^ Advance care planning for people who have attended emergency departments frequently should be co-produced, should never be used to exclude a person from treatment, and must include a preventive care plan as well as a response plan if it is to be effective.^8^ Mental health flagging should be used cautiously: in some settings it has been found to harm rather than help.^9^ In reality, each occasion offers an opportunity to engage, to support, to advocate, to challenge stigma and discrimination, and to play one's part in establishing a trusting, compassionate and respectful relationship with healthcare services.

### Doctor's perspective

You go to see the patient as soon as you can. She is clearly distressed, still coughing up blood, asking staff to leave her alone and repeatedly saying ‘I need to go, it's all contaminated’. The medical team recommend a chest X-ray to assess haemoptysis, and transfer to the burns unit. Although she is in no state to give a coherent psychiatric history, she is clearly in need of help and your emergency department colleagues are reassured that you have come to assist. You offer her lorazepam and she declines, saying ‘I need to go, I'm hurting people’. You assess her capacity to decide whether to stay or go and conclude that she cannot currently use or weigh information about her health, and that it is in her best interests to stay for emergency medical treatment. By remaining with her to explain what is happening, listening to her concerns and encouraging your emergency department colleagues to be patient, you are able to gradually help her calm down sufficiently to accept medical attention without restraining or sedating her.

### Patient's perspective

*Turning up to advise the medical staff and to help me makes a huge difference. When I'm worried about contamination and in a cycle of compulsions, being in the emergency department is terrifying. ‘Medically cleared’ is unhelpful: if I leave hospital as soon as I can for fear that I am contaminating and killing the staff and other patients, there will never be a point where I become sufficiently ‘medically cleared’ to receive psychiatric help. There is an element of immediacy in what is needed, in guiding medical staff to help and to consider my capacity. Looking back now at the injuries I have and how they have affected my ability to work in my profession, I wish more had been done to understand how much I could weigh information when I had serious injuries, but I was also feeling I needed to decline treatment and leave because I was contaminating people*.

### Doctor's perspective

On mental state examination, she has repetitive intrusive thoughts that she has unintentionally hurt or killed people, and a deeply unpleasant crawling sensation just beneath her skin, which she believes is an unexplained contamination that can spread to others without physical contact. The purpose of chemically burning her skin is not to address emotional distress, nor to inflict pain or injury, but to neutralise this contamination, thus ensuring the safety of others. She describes the risk of dying or losing a limb as ‘collateral’ for ensuring others are safe. On systematic enquiry, you identify that she eats minimally and only from sealed packets for fear that food is contaminated. She spends long periods walking or running outdoors until she has reached a ‘safe’ (prime) number of miles. She has significant anxiety associated with non-prime numbers, which feel ‘unsafe’ and may somehow harm others. In the past she has deleted entire dissertations and research manuscripts before submission because she feared that making a mistake might cause people to die. It is clear from the absence of emotional dysregulation and her history of stable long-term relationships in the social domains of professional work, friendship and personal relationships that she does not have a personality disorder. It is also clear from the absence of hallucinations and the fact that her fears are obsessional rather than delusional that she does not have a psychosis. Therefore you believe that she has obsessive–compulsive disorder (OCD).

Question 2: What is your role in this scenario?

(a) Advising medical staff on capacity to make decisions about medical treatment.
(b) Diagnostic assessment alongside assessment of needs and risk.
(c) Explaining the nature of OCD to the patient, offering hope and ensuring she can access effective treatment following discharge.
(d) All of the above.

Answer: (d)

## Severity of OCD

Severe mental illness is not defined by diagnosis but by the degree of distress, disability and interference in a person's life. Without treatment, OCD can be severe, disabling and enduring, with major effects on physical health; as well as self-harming, people with OCD may restrict fluid or food intake because of either neglect or obsessions about contamination. Others may have excessive slowness or be ruminating all day and unable to function. Although most people respond to community-based treatment, some people with OCD may need admission to a psychiatric ward to engage in specialist cognitive–behavioural therapy (CBT) and for supervision of pharmacotherapy. The World Health Organization has classified OCD among the top 10 most disabling illnesses in terms of lost income and decreased quality of life.^10^ People with OCD may experience significant delays to diagnosis, both from a fear of asking for help and also from delays in healthcare staff identifying the condition.^11^ One study found that the average time to receiving first treatment for OCD was more than 17 years from the onset of first symptoms, and more than 11 years after fully meeting diagnostic criteria.^12^

## OCD and self-harm

In clinical practice, self-harm may be erroneously viewed as always being a means of coping with emotional distress. Self-harm accompanies a wide range of psychiatric disorders, including psychotic, neurodevelopmental, affective, anxiety and personality disorders. People who have self-harmed sometimes encounter prejudice and discrimination from healthcare staff, which inhibits access to effective assessment and treatment.^13,14^ A key issue in formulation and diagnosis is understanding the circumstances, precipitants, intention and motivation behind the self-harm.

Despite commonly experiencing ego-dystonic obsessional fears about causing harm, people with OCD rarely harm others.^15^ On the other hand, self-harm is common in OCD, with an estimated prevalence of 7.3%, and takes many forms^16^; it is usually ego-dystonic and compulsive in nature. The manifestations can be encountered by many other specialties, especially dermatology; it has been estimated that between 9 and 35% of patients with OCD will present for treatment of complications related to skin damage.^17^ Compulsive washing or decontamination with irritant substances such as disinfectants or bleach are among the most commonly recognised compulsions, and people with OCD may present with atopic dermatitis, irritant toxic dermatitis or dry skin eczema.^18^ Compulsive self-cutting and decontamination by chemical burning are less common; understanding the intention behind the behaviour is important in making the diagnosis.

Excoriation disorder (skin-picking or dermatillomania) is relatively frequent, with a prevalence between 1.4 and 5.4%;^19,20^ it has gained increasing attention and has been defined as a specific type of obsessive–compulsive and related disorders in the DSM-5 and the proposed ICD-11.^21^ Excoriation disorder can lead to serious complications including infection, physical disfigurement and physical disability.^22,23^ Trichotillomania (hair pulling disorder) has a point prevalence of 0.5–2.0% and predominantly affects female patients;^24^ similar to excoriation disorder, it has been identified as an OCD type. Rarely, self-surgery such as autocastration has been described as a complication of OCD.^25^

## OCD and suicide

It is important to recognise that people with OCD are at increased risk of suicide.^26^ A Swedish population-based study found that suicide attempt rates were five times higher and suicide mortality rates ten times higher in people with OCD compared with the general population.^27^ A systematic review found median rates of suicidal ideation and suicide attempts of 27.9% and 10.3%, respectively, in people with OCD.^23^ Comorbid conditions such as harmful or dependent use of alcohol or other drugs, personality disorders or affective disorders increase suicide mortality rates in OCD to between 40 and 82%; however, the risk remains high when OCD is the only condition present.^22,28^

## Assessment of obsessions and compulsions

Obsessions are thoughts which come into one's head over and over again, and will not go away. Often experienced as paralysing and terrifying, they can be about apparently mundane things, such as the idea that something is not clean or that an appliance has been left on. They can also be more obviously upsetting, such as believing that one might stab someone (despite not wanting to) or might have unintentionally killed or harmed someone. Inappropriate sexual thoughts may take a similar form but be difficult to disclose in view of the associated shame. Obsessions can be about one's own body – for instance, having an infectious disease or something dangerous under the skin – and can be associated with intense physical sensations such as a visceral feeling of disgust, crawling skin, feeling contaminated or unclean, or intense physical anxiety or shame.

As well as physical contamination from direct contact with dirt or perceived contaminants, a person may experience mental contamination – feelings and fears that arise without physical contact.^29^ The source of contamination is human rather than inanimate, and the feelings of dirtiness may come from the individual with OCD. Mental contamination is often associated with another person having abused, betrayed or humiliated the patient. The self may be regarded as ‘bad’ or ‘immoral’, and the essence of this badness may be transferred to objects and then passed to others. In this case, the patient's motivation is to decontaminate her ‘self’ to prevent harm being transferred to others; she also has an over-inflated sense of responsibility and influence in believing herself responsible for this harm.^30^ Compulsive washing is often less effective in mental contamination, hence in this case the escalation to chemical burning.

Obsessions can follow convoluted paths to extreme consequences. For example, a worry that a light was not turned off might lead to obsessions that a spark of electricity could start a fire and kill people inside the building. A worry that one has left the door unlocked may lead to obsessions that a murderer could break into the house and kill one's family. Common features of obsessions are is that they are unpleasant, upsetting and cannot be ignored. A key feature is the recognition that these ideas are not correct, do not make sense or are about something which the person does not want to do; they are ‘ego-dystonic’. A patient may say that the rational part of their brain can see that it does not make sense, but that does not stop them from experiencing intense fears that the thought may be true.

Compulsions are repetitive, purposeful physical or mental actions that the individual feels compelled to engage in according to rules or until it feels ‘safe’, ‘comfortable’ or ‘just right’, in order to quell the anxiety, fear, disgust or terror associated with an obsession. Compulsions can involve checking, touching, arranging, decontamination, walking, counting or other physical actions. Alternatively, compulsions may involve mental actions such as praying, reciting or making number patterns. Compulsions are linked to obsessions in that they are used to try to get rid of them or fill the need they create. Compulsions can be resisted temporarily or deferred but almost always end up being performed, as the distress from not doing them is great and continuous. They feel voluntary to the person; they are not being controlled. This means people with OCD often blame themselves or are seen as acting irrationally but with capacity by healthcare staff. Although performing compulsions leads to temporary relief of distress, in the longer term it maintains distress by reinforcing the need to act to seek relief.

When differentiating obsessions, ruminations, delusions and thought interference, it is important to enquire about the nature of the thought. Some people with severe OCD refer to obsessions as ‘voices’ or speak about compulsions as though they are being ordered; it is important not to assume that these are command hallucinations without detailed examination of phenomenology. In addition to asking whether a patient recognises thoughts as their own, ask how easy it is to distract themselves, whether the thoughts are repeatedly intrusive or ruminative, and whether they fear worse consequences if they do not perform a certain act. When enquiring about compulsions, ask what will happen if the patient doesn't do the action, and how they feel once they have done the action.^31^ Becoming familiar with a symptom checklist such as the Yale–Brown Obsessive–Compulsive Scale will help in developing a systematic approach to enquiry about obsessions and compulsions.^32^

## Practical management of OCD

When someone is in the midst of terrifying obsessions, calm listening and explanation will help the obsessions and associated anxiety pass. If severe, this may take several hours. In an emergency situation, anxiolytic drugs may help to alleviate anxiety and allow a person to accept medical care, although they may also cause disinhibition and exacerbate compulsions. Anxiolytics should not be used routinely for obsessions outside emergencies.

Although there is discussion about the pros and cons of diagnosis in some psychiatric conditions, OCD is a condition where diagnosis allows for a clear explanation and treatment plan. When meeting a patient whom you suspect has OCD, it is important to confirm the diagnosis; ask for senior help if you are not sure. Once confirmed, you can give the patient hope: explain that it is a treatable condition, give written information about self-help, and ensure that follow-up and access to effective treatment are available. If OCD is identified while on call and an immediate management plan made, the patient should be handed over to the liaison psychiatry team for ongoing support in hospital, initiation or review of drug treatment, and arrangement of appropriate aftercare.

It is essential to ensure that appropriate follow-up and treatment are arranged, including specialist CBT for OCD that includes exposure and response prevention (ERP). Longer-term treatment should follow the recommendations in the National Institute for Health and Care Excellence guidelines:^33^ treatment with CBT for OCD that includes ERP, plus the maximum tolerated dose of two trials of selective serotonin reuptake inhibitors or clomipramine for at least 12 weeks each. If a patient still has clinically significant symptoms interfering with functioning, a multidisciplinary review should be undertaken, and the patient should be referred to a multidisciplinary team with specific expertise in the treatment of OCD for assessment and further treatment planning, including augmenting drug treatment and intensive CBT for OCD.

## Reflections and considerations

We have used the lived experience of OCD, self-harm and mental health services to illustrate a scenario which may be encountered by mental health staff working on call in emergency departments. This highlights several learning points about OCD, self-harm and on-call working. When on call, working collaboratively alongside other medical specialties is of benefit to patients. When seeing people who have self-harmed, retaining compassion, curiosity and hope for change, and ensuring a skilled assessment every time, can improve both patient experience and clinical outcomes. The recognition and diagnosis of OCD are essential elements of mental health staff's clinical skills; identifying and treating OCD can alleviate significant suffering and disability, and can save lives.

## Patient's reflections and considerations

*What matters to me in the emergency department isn't just about ‘assessment’ but is about providing help. There is a longer-term element in considering my ability to recover and to continue working: ensuring that I receive effective long-term treatment for OCD. Being correctly diagnosed with OCD has been life-saving for me. Until that point I was caught in a cycle of obsessions and compulsions which were causing me so much harm as to become life-threatening. I couldn't see a way out other than taking my own life to prevent me hurting or killing other people, but the correct diagnosis has improved things in ways I wouldn't have believed were possible. In the 4 years up to that point I had been brought to the emergency department over 133 times. I nearly died and was ventilated in intensive care after taking overdoses. The police arrested and prosecuted me when I was suicidal and afraid because I couldn't get the right help, and the chemical burns which I believed were necessary to stop the contamination spreading and killing others have caused permanent physical disability, ending my career.*^34,35^ *In the 2 years since I received the right diagnosis, explanation and drug treatment, although I am still waiting for specialist cognitive behaviour therapy and still spend hours every night terrified I have killed people, I have completely stopped compulsive chemical burning, I have not tried to kill myself, I have not been brought back to the emergency department at all and I have developed an alternative career*.

*In this context I am the patient, but I've been called worse: ‘Frequent Flyer’, ‘A Waste of Valuable Clinical Time and Resources’, ‘Very Clever and Manipulative’ (written in my notes while I was unresponsive in resus following an overdose), ‘That’ (as in, ‘I'm not touching That’). Self-harm, particularly repeat self-harm, attracts stigma that is unacceptable, along with stereotypes and assumptions which can distract from the clinical picture. What I need from you is not only your clinical expertise, but your clinical leadership in modelling respect for me and challenging discriminatory behaviour. Your work may be hard, but it is crucial; the difference your attitude and assistance makes can be life-saving and life-changing*.

## About the authors

**Erika Palombini** is a Core Specialty Registrar in Liaison Psychiatry at the Department of Psychological Medicine, Northwick Park Hospital, Central and North West London NHS Foundation Trust, UK. **Joel Richardson** is a Foundation Doctor in Liaison Psychiatry at the Department of Psychological Medicine, Northwick Park Hospital, Central and North West London NHS Foundation Trust, UK. **Emma McAllister** is a Lived Experience Advisor, UK. **David Veale** is a Consultant Psychiatrist in Cognitive Behaviour Therapy at South London and Maudsley NHS Foundation Trust, and Visiting Professor at the Institute of Psychiatry Psychology and Neuroscience, King's College London, UK. **Alex B. Thomson** is a Consultant Liaison Psychiatrist at the Department of Psychological Medicine, Northwick Park Hospital, Central and North West London NHS Foundation Trust, UK.
